# Effects of *cpxR* on the growth characteristics and antibiotic production of *Xenorhabdus nematophila*

**DOI:** 10.1111/1751-7915.13362

**Published:** 2019-01-08

**Authors:** Shuqi Guo, Zeyu Wang, Beiling Liu, Jiangtao Gao, Xiangling Fang, Qian Tang, Muhammad Bilal, Yonghong Wang, Xing Zhang

**Affiliations:** ^1^ Research and Development Center of Biorational Pesticides Key Laboratory of Plant Protection Resources and Pest Management of Ministry of Education Northwest A&F University 22 Xinong Road Yangling Shaanxi 712100 China; ^2^ State Key Laboratory of Microbial Metabolism School of Life Sciences and Biotechnology Shanghai Jiao Tong University Shanghai 200240 China; ^3^ School of Food and Biological Engineering Shaanxi University of Science & Technology Weiyang University Campus Xi ‘an Shaanxi 710021 China; ^4^ State Key Laboratory of Grassland Agro‐ecosystems College of Pastoral Agriculture Science and Technology Lanzhou University Lanzhou 730020 China; ^5^ School of Agriculture and Environment Faculty of Science The University of Western Australia 35 Stirling Highway Crawley WA 6009 Australia; ^6^ School of Life Science and Food Engineering Huaiyin Institute of Technology Huaian 223003 China; ^7^ Shaanxi Research Center of Biopesticide Engineering and Technology Northwest A&F University 22 Xinong Road Yangling Shaanxi 712100 China

## Abstract

CpxR is a global response regulator that negatively influences the antimicrobial activities of *Xenorhabdus nematophila*. Herein, the wildtype and Δ*cpxR* mutant of *X. nematophila* were cultured in a 5‐l and 70‐l bioreactor. The kinetic analysis showed that Δ*cpxR* significantly increased the cell biomass and antibiotic activity. The maximum dry cell weight (DCW) and antibiotic activity of Δ*cpxR* were 20.77 ± 1.56 g L^−1^ and 492.0 ± 31.2 U ml^−1^ and increased by 17.28 and 97.33% compared to the wildtype respectively. Xenocoumacin 1 (Xcn1), a major antimicrobial compound, was increased 3.07‐fold, but nematophin was decreased by 48.7%. In 70‐l bioreactor, DCW was increased by 18.97%, while antibiotic activity and Xcn1 were decreased by 27.71% and 11.0% compared to that in 5‐l bioreactor respectively. Notably, pH had remarkable effects on the cell biomass and antibiotic activity of Δ*cpxR*, where Δ*cpxR* was sensitive to alkaline pH conditions. The optimal cell growth and antibiotic activity of Δ*cpxR* occurred at pH 7.0, while Xcn1 was increased 5.45‐ and 3.87‐fold relative to that at pH 5.5 and 8.5 respectively. These findings confirmed that Δ*cpxR* considerably increased the biomass of *X. nematophila* at a late stage of fermentation. In addition, Δ*cpxR* significantly promoted the biosynthesis of Xcns but decreased the production of nematophin.

**Abbreviations**
$\bar{\mu }$: average specific cell growth rate (h^−1^)
$\bar{q_{P}}$: average specific antibiotic production rate (U mg^−1^ h^−1^)
$\bar{q_{s}}$: average specific glucose consumption rate (h^−1^)
DCW: dry cell weight (g L^−1^)
DO: dissolved oxygen (%)
*P*_max_: maximal antibiotic activity (U ml^−1^)
*P*_P_: antibiotic productivity (U ml^−1^ h^−1^)
*P*_X_: cell production rate (g L^−1^ h^−1^)
*q*_p_: specific antibiotic production rate (U mg^−1^ h^−1^)
*q*_s_: specific glucose consumption rate (h^−1^)
*X*_max_: maximal cell density (g L^−1^)
*Y*_P/S_: antibiotic yield of glucose (U g^−1^)
*Y*_P/X_: antibiotic yield on the cell (U g^−1^)
*Y*_X/S_: cell yield on glucose (g g^−1^)
*μ*_max_: maximal specific cell growth rate (h^−1^)
*μ*: specific cell growth rate (h^−1^)

## Introduction

*Xenorhabdus nematophila* is a unique Gram‐negative bacterium that develops a mutualistic association with an infective dauer juvenile (IJ) insect‐pathogenic nematode in the genus of *Steinernema* (Thomas and Poinar, 1979). Recent research has revealed *X. nematophila* to be a rich source for natural products with potent biological or pharmaceutical activities (Challinor and Bode, 2015; Bozhüyük *et al*., 2016; Tobias *et al*., 2017). In *X. nematophila* ATCC 19061, 7.5% of the genome encoding proteins is involved in secondary metabolism; however, the majority of these encoded molecules are cryptic (Chaston *et al*., 2011). Until now, *X. nematophila* has been known to produce several secondary metabolites, including indole derivatives (Paul *et al*., 1981; Sundar and Chang, 1993; Li *et al*., 1995), nematophin (Li *et al*., 1997), insecticidal proteins (Morgan *et al*., 2001; Sergeant *et al*., 2003; Yang *et al*., 2009; Sheets *et al*., 2011; Hinchliffe, 2010), benzylideneacetone (Ji *et al*., 2004), xenocoumacins (Xcns) (McInerney *et al*., 1991) and the major class of non‐ribosomal produced secondary metabolites (Crawford *et al*., 2011). Due to structural diversity, these metabolites display a wide range of bioactivities with potential interest for pharmaceutical, agricultural and chemical applications. These naturally occurring antibiotics and their derivatives may provide beneficial leads in the future research and manufacturing of agrochemicals.

Nematophin is a 3‐indoleethyl 3′‐methyl‐2′‐oxopentanamide consisting of an N‐terminal α‐keto acid and a C‐terminal tryptamine (TRA), first isolated from strain *X. nematophila* BC1. It displays strong antifungal and antibacterial activities (Li *et al*., 1997; Kennedy *et al*., 2000). A monomodular non‐ribosomal peptide synthetase (NRPS), RdpD, is responsible for the biosynthesis of nematophin (Cai *et al*., 2017). Xenocoumacins, including Xcn1 and Xcn2, are the principal antimicrobial compounds secreted by *X. nematophila*. Xcn1 exhibits marked antimicrobial activity against Gram‐positive bacteria and fungal strains, while Xcn 2 shows substantially reduced bioactivity (McInerney *et al*., 1991; Huang *et al*., 2005, 2006; Yang *et al*., 2011; Zhou *et al*., 2017). Meanwhile, it has been demonstrated that fourteen genes (*xcnA‐xcnN*) are involved in the synthesis of Xcns in *X. nematophila*. The genes *xcnA‐L* are responsible for Xcn1 synthesis, whereas the genes *xcnM* and *xcnN* control the conversion of Xcn1 into Xcn2 (Park *et al*., 2009). A prominent increase in Xcn1 along with a 20‐fold reduced cell viability following attenuated Xcn 2 production suggests that the conversion of Xcn 1 to Xcn 2 is a resistance mechanism to circumvent self‐toxicity (Park *et al*., 2009).

The rapid bacterial adaptation ability to changing environmental conditions is crucial for their growth and pathogenicity. Reports have shown that the growth of *X. nematophila* occurs in the nematode vesicle, a stressful and nutrient‐deficient environment. Nevertheless, *X. nematophila* is speculated to encounter a relatively nutrient‐rich environment, when released from nematode vesicles to insect haemolymph (Goodrich‐Blair and Clarke, 2007; Herbert and Goodrich‐Blair, 2007). In response to nutrient upshift, the bacteria grow rapidly and produce several metabolites to overcoming the insect immune system (Forst and Nealson, 1996) and preventing the growth of various bacterial and fungal competitors (Akhurst, 1982; Chen *et al*., 1994). Several reports confirmed that the antimicrobial activity of *X. nematophila* varies according to the environment conditions (Chen *et al*., 1996; Wang *et al*., 2008a,b, 2010, 2011). However, it remains unclear how *X. nematophila* recognizes these changes or how these signals are associated with the antimicrobial activity. Increasing evidence indicates that two two‐component systems (CpxRA and EnvZ/OmpR) are involved in responding to both of the nematode and insect environments (Park and Forst, 2006; Herbert *et al*., 2007; Tran and Goodrich‐Blair, 2009; Tran and Goodrich‐Blair, 2009). As a sensor histidine kinase, CpxA and EnvZ phosphorylate its cognate response regulator CpxR and OmpR in response to change in pH and osmolality as well as other stimuli and regulate key genes essential for establishing interactions with each of these hosts.

As a global regulator, OmpR also represses antibiotic production, in Δ*ompR* mutant strain, Xcn1 level and expression of *xcnA–L* were increased, while Xcn2 level and *xcnMN* expression were decreased (Park and Forst, 2006; Park *et al*., 2009). Interestingly, CpxR also exhibits a similar regulatory mechanism for Xcns biosynthesis as OmpR. In Δ*cpxR* mutant strain, the levels of *xcnA–L* expression were amplified, while *xcnMN* expression was diminished (Tang, 2015). However, to date, there is no report concerning the effects of CpxR on the growth characteristics and antibiotics biosynthesis potential of *X. nematophila*. Therefore, the present study investigated the effects of CpxR on the antibiotics production an growth of *X. nematophila*. The kinetics analysis of batch fermentation of the wildtype and Δ*cpxR* mutant of *X. nematophila* YL001 was carried out in a 5‐l laboratory‐scale bioreactor. Specifically, we tested whether CpxR influenced the production of nematophin and Xcns.

## Results

### Effects of *cpxR* disruption on cell growth and antibiotics production

**Figure 1:**
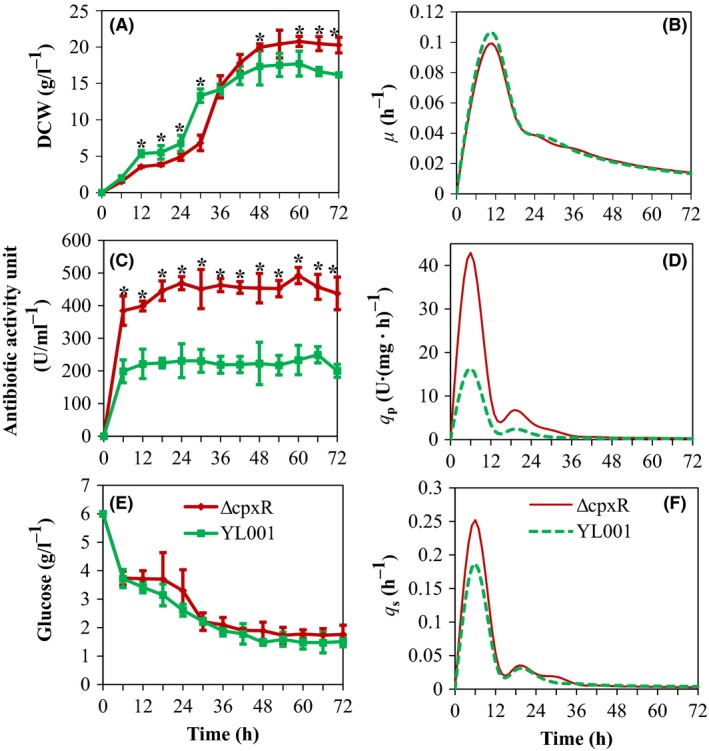
Time profiles of wildtype and Δ*cpxR* mutant of *Xenorhabdus nematophila* YL001 during cultivation in the 5‐l bioreactor. A. Cell growth B. Specific cell growth rate (*μ*). C. Antibiotic activity. D. Specific antibiotic production rate (*q*_p_). E. Glucose. F. Specific glucose consumption rate (*q*_s_). Error bars indicate standard deviations from three independent experimental measurements. Asterisk symbols denote significant difference at *P *=* *0.05 at different time between wildtype and Δ*cpxR* mutant of *Xenorhabdus nematophila* YL001.

**Figure 2:**
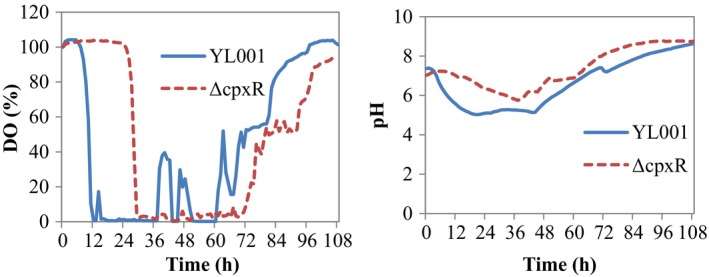
The variations in dissolved oxygen concentration and pH of wildtype and Δ*cpxR* mutant of *Xenorhabdus nematophila* YL001 during cultivation in the 5‐l bioreactor.

The batch fermentation kinetics of the wildtype and Δ*cpxR* of *X. nematophila* YL001 were studied in a 5‐l bioreactor. Evidently, a distinct exponential and stationary phase was observed from the bacterial growth curve (Fig. 1A). During 0–36 h of the bioprocess, the DCW of the wildtype strain was higher than that of Δ*cpxR*; however, after 36 h, the DCW of Δ*cpxR* was found to be higher in comparison with the wildtype strain. Due to the rapid growth of wildtype compared with Δ*cpxR* at earlier fermentation stage, DO concentration and pH decreased rapidly (Fig. 2). The maximum DCW of the wildtype and Δ*cpxR* showed an optimal value of 17.71 ± 1.35 and 20.77 ± 1.56 g L^−1^, respectively, when fermentation proceeded to the stationary phase, where the DO concentration was 17.8 and 3.5% respectively (Figs 1A and 2). However, the maximum specific cell growth rate (*μ*_max_) of Δ*cpxR* was 0.099 ± 0.007 h^−1^ and found to be lower compared with the wildtype strain (Fig. 1B). The cell yields on glucose (*Y*_X/S_) and cell production rate (*P*_X_) of Δ*cpxR* were higher than that of the wildtype strain (Table 1). It is obvious that Δ*cpxR* mutation favoured the cell production. In consonance with diminishing cell biomass of Δ*cpxR* during 0–36 h, the consumption of glucose was declined.

**Table 1:** Batch fermentation parameters of wildtype and Δ*cpxR* of *Xenorhabdus nematophila* YL001at pH 7.0 in 5‐ and 70‐l bioreactor

Parameters	5 L		70 L
	YL001	Δ*cpxR*	Δ*cpxR*
*X*_max_ (g L^−1^)	17.71 ± 1.35	20.77 ± 1.56^a^	24.71 ± 2.31^b^
*P*_max_ (U ml^−1^)	249.33 ± 15.23	492.0 ± 31.20^a^	381.07 ± 15.74^b^
*P*_*X*_ (g L^−1^ h^−1^)	0.30 ± 0.05	0.35 ± 0.03	0.41 ± 0.06^b^
*P*_*P*_ (U ml^−1^ h^−1^)	3.78 ± 0.28	8.20 ± 1.20^a^	9.07 ± 2.53
*μ* (h^−1^)	0.107 ± 0.02	0.099 ± 0.007	0.035 ± 0.003^b^
$\bar{\mu }$ (h^−1^)	0.037 ± 0.003	0.036 ± 0.004	0.021 ± 0.002^b^
*q*_*p*_ (U mg^−1^ h^−1^)	16.35 ± 1.23	42.87 ± 3.67^a^	6.410 ± 0.89^b^
$\bar{q_{p}}$ (U mg^−1^ h^−1^)	2.29 ± 0.26	6.04 ± 1.25^a^	1.348 ± 0.26^b^
*Y*_*X/S*_ (g g^−1^)	2.82 ± 0.08	3.05 ± 0.55^a^	5.626 ± 1.06^b^
*Y*_*P/S*_ (U g^−1^)	55.05 ± 6.21	116.52 ± 9.35^a^	87.32 ± 12.3
*Y*_*P/X*_ (U g^−1^)	14.99 ± 1.36	23.69 ± 0.89^a^	16.44 ± 1.42^b^
*q*_s_(h^−1)^	0.187 ± 0.06	0.252 ± 0.03^a^	0.132 ± 0.002^b^
$\bar{q_{s}}$	0.028 ± 0.004	0.036 ± 0.002^a^	0.024 ± 0.003^b^

Data are presented as a mean ± standard deviation from three replicated experiments.

a Followed by the data in the column indicates significant differences of Δ*cpxR* and YL001 in 5‐l bioreactor at *P *=* *0.05.

b Followed by the data in the column indicates significant differences of Δ*cpxR* between fermentation in 5‐ and 70‐l bioreactor at *P *=* *0.05.

During the bioprocess, Δ*cpxR* showed a significantly higher (*p *<* *0.05, *t *=* *43.33, *d*_f _= 10) antibiotic activity (Fig. 1C). The maximum antibiotic activity (492.0 ± 31.2 U ml^−1^) of Δ*cpxR*, which was increased by 97.33% compared with the wildtype (249.33 ± 15.23 U ml^−1^), was achieved at 60 h. Profiles of the specific antibiotic production rate (*q*_p_) of the wildtype strain and Δ*cpxR* showed identical trends. The time duration to reaching the maximum *q*_p_ was similar, whereas the declining rate was found to be different after *q*_p_ reached the maximum (Fig. 1D). The maximum *q*_p_ of the wildtype and Δ*cpxR* was 16.35 ± 1.23 and 42.87 ± 3.67 U mg^−1^ h^−1^ respectively. During the bioprocess, the average specific antibiotic biosynthesis rate of the wildtype and Δ*cpxR* was 6.04 ± 1.25 and 2.29 ± 0.26 U mg^−1^ h^−1^ respectively. Similarly, the optimum antibiotic yield on glucose (*Y*_P/S_), the antibiotic productivity (*P*_P_) and the optimum antibiotic yield on cell (*Y*_P/X_) of Δ*cpxR*, which were 116.52 ± 9.35 U g^−1^, 8.20 ± 1.20 U ml^−1^ h^−1^ and 23.69 ± 0.89 U g^−1^, respectively, were higher than that of the wildtype strain (Table 1). It can be concluded that Δ*cpxR* could significantly increase the antibiotic activity of *X. nematophila*.

**Figure 3:**
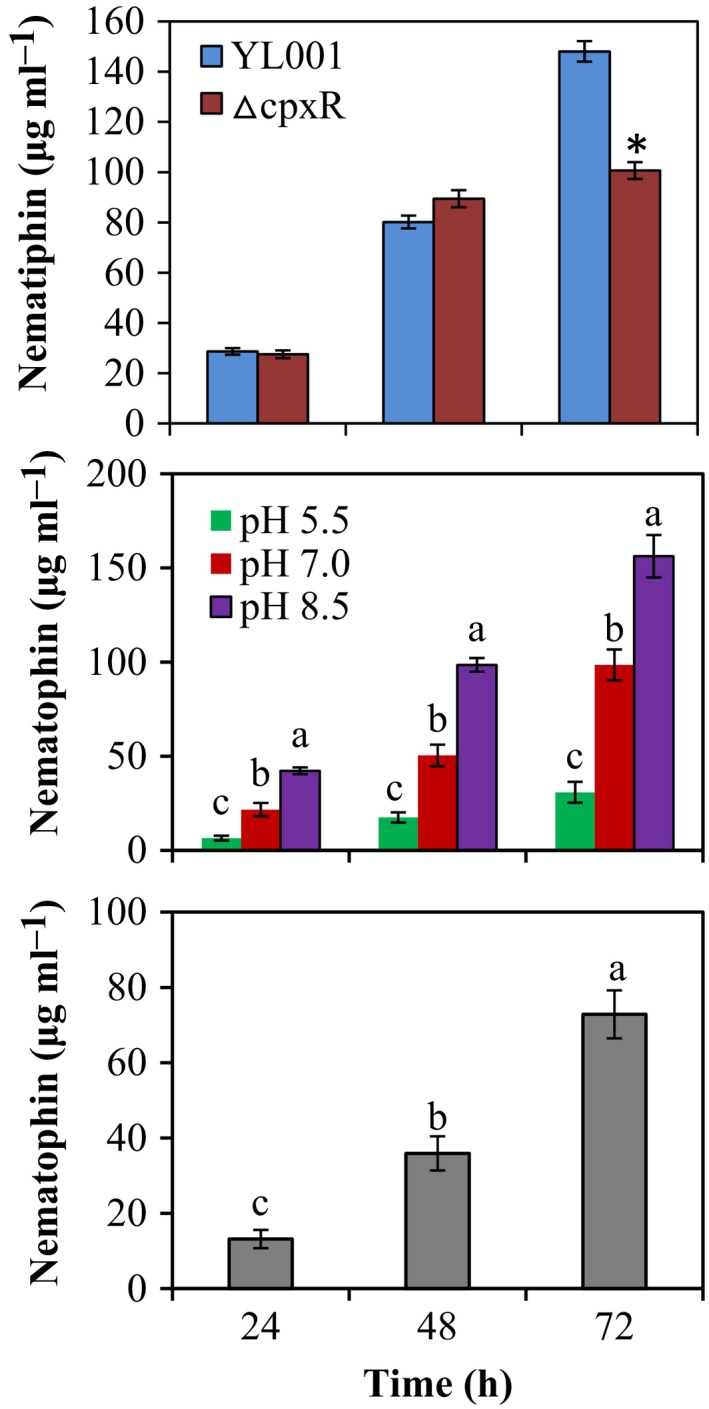
The concentration of nematophin produced by wildtype and Δ*cpxR* mutant of *Xenorhabdus nematophila* YL001. A. The wildtype and Δ*cpxR* mutant were cultured at pH 7.0 in 5‐l bioreactor. B. Δ*cpxR* mutant was cultured at pH 5.5, 7.0 and 8.5 in 5‐l bioreactor. C. Δ*cpxR* mutant was cultivated at pH 7.0 in 70‐l bioreactor. Error bars indicate standard deviations from three independent samples. Different lowercase letters above the bar indicate significant differences at *P *=* *0.05. Asterisk symbols denote significant difference at *P *=* *0.05.

Based on our findings that Δ*cpxR* mutant exhibited higher antibiotic activity against *B. subtilis*, we also investigated the variations of nematophin and Xcns. At 24 and 48 h of the bioprocesses, Δ*cpxR* had no obvious (*p *>* *0.05, *t *=* *1.22, *d*_f_ = 4, 24 h; *t *=* *−2.20, *d*_f_ = 4, 48 h) effect on nematophin production, but at 72 h, the levels of nematophin in Δ*cpxR* were significantly decreased by 48.7% compared with the wildtype strain (Figs 3A and S1). In addition, *Y*_*P/S*_, *P*_*P*_. *Y*_*P/X*_ and *q*_p_ of nematophin in Δ*cpxR* were decreased by 41.12, 32.04, 45.68 and 45.67%, respectively, compared with the wildtype strain (Table 2). However, Xcn1 in Δ*cpxR* was 6.41‐fold greater in contrast with the native strain (Fig. S2). These results indicate that CpxR positively regulates the production of nematophin while negatively regulates Xcns synthesis.

**Table 2:** Parameters of the batch fermentation of nematophin produced by *Xenorhabdus nematophila* YL001 wildtype and Δ*cpxR* at different pH in 5‐ and 70‐l bioreactor

Parameters	5 L				70 L
	YL001	Δ*cpxR*			Δ*cpxR*
	pH 7.0	pH 5.5	pH 7.0	pH 8.5	pH 7.0
Nematophin (μg ml^−1^)	148.06 ± 5.61 a	42.30 ± 3.67 c	100.67 ± 3.38 b	156.2 ± 3.67 a	72.85 ± 2.58*
*P*_*P*_ (μg ml^−1^ h^−1^)	2.06 ± 0.35 a	0.59 ± 0.12 c	1.40 ± 0.26 b	2.17 ± 0.18 a	1.01 ± 0.03*
*q*_p_ (μg mg^−1^ h^−1^)	0.127 ± 0.015 a	0.042 ± .011 c	0.069 ± .0012 b	0.135 ± .005 a	0.042 ± .012*
*Y*_P/S_ (μg g^−1^)	32.98 ± 2.58 b	9.86 ± 1.67 d	19.42 ± 2.31c	36.26 ± 0.58 a	16.70 ± 0.37*
*Y*_*P/X*_ (μg g^−1^)	9.15 ± 1.32 a	3.02 ± 0.85 c	4.97 ± 0.78 b	9.69 ± 1.35 a	3.04 ± 0.79*

Data are presented as a mean ± standard deviation from three replicated experiments. Different lowercase letters followed by the data in each row indicate significant differences at *P *=* *0.05. ‘*’ followed by the data in the column indicates significant differences of Δ*cpxR* fermentation in pH 7.0 between in 5‐ and 70‐l bioreactor at *P *=* *0.05.

The rapid growth and increase in biomass of the wildtype at an early phase of fermentation resulted in the rapid decrease in DO concentration compared with Δ*cpxR* (Fig. 2). Correspondingly, glucose uptake of the wildtype was faster than Δ*cpxR* (Fig. 1E). At the late stage of fermentation, an increase in biomass of Δ*cpxR* led to decreased DO concentration, but the uptake of glucose between two strains had no significant difference (Fig. 1E). At the end of the fermentation, the glucose concentration in the broth of the strains was 1.51 and 1.76 g L^−1^, and the total glucose consumption was 74.8 and 70.67%. Profiles of the specific glucose consumption rate (*q*_s_) of the wildtype and Δ*cpxR* displayed comparable trends. The time duration of attaining the maximum *q*_s_ was identical, but a different decaying trend was observed after *q*_s_ reached the maximum (Fig. 1F). Notably, the maximum *q*_s_ and the average specific glucose consumption rate of the wildtype and Δ*cpxR* showed a significant difference (Table 1).

### Effects of pH on the cell growth and antibiotics production of Δ*cpxR*

**Figure 4:**
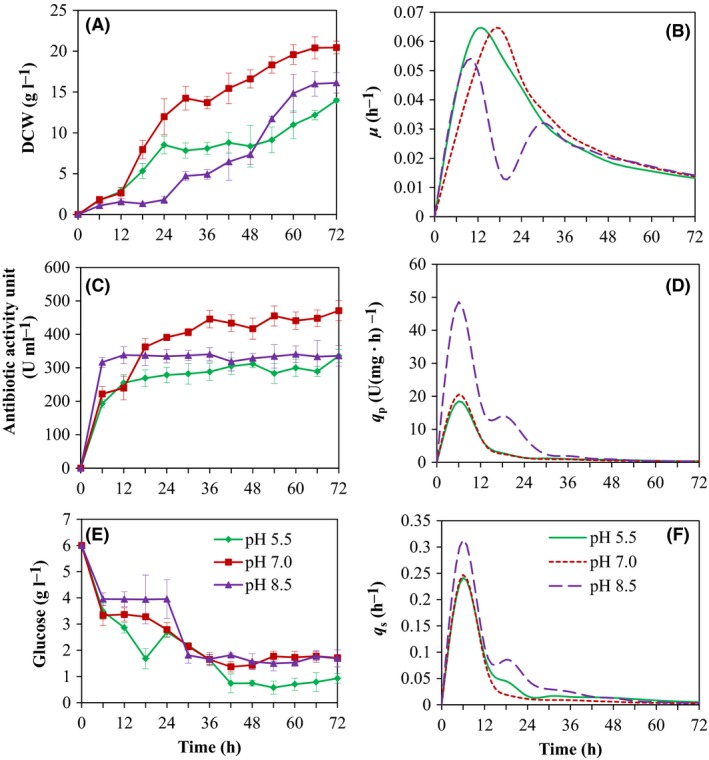
Time profiles of Δ*cpxR* mutant of *Xenorhabdus nematophila* YL001 at different pH during cultivation in the 5‐l bioreactor. A. Cell growth. B. Specific cell growth rate (*μ*). C. Antibiotic activity. D. Specific antibiotic production rate (*q*_p_). E. Glucose. F. Specific glucose consumption rate(*q*_s_).

**Figure 5:**
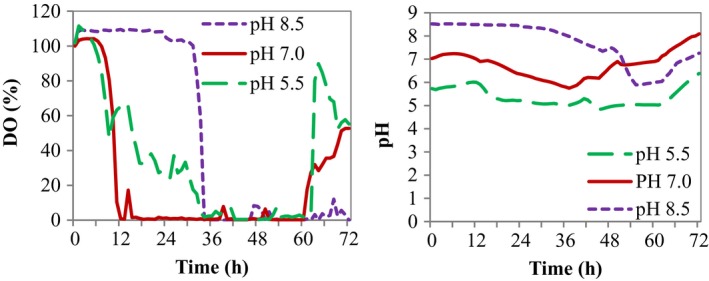
The variations in dissolved oxygen concentration and pH of Δ*cpxR* mutant of *Xenorhabdus nematophila* YL001 during cultivation at different pH in the 5‐l bioreactor.

At different initial pH, cell growth was observed to be considerably changed with the pH operation applied (Fig. 4A). At pH 8.5, the presence of an obvious lag phase was characterized by a higher DO concentration at an early phase of fermentation (Fig. 5). During 0–42 h of the fermentation, the DCW was reduced at pH 8.5 in comparison with other pH conditions. After 48 h, the DCW was observed to be higher than that at pH 5.5 and lower than that at pH 7.0. The highest DCW of each original pH displayed an optimum value of 20.44 ± 2.65 g L^−1^ at pH 7.0, which was reduced by 31.5% and 21.14% at pH 5.5 and 8.5 respectively. The maximum specific cell growth rate (*μ*_max_) at pH 5.5, 7.0 and 8.5 was 0.065 ± 0.013, 0.065 ± 0.009 and 0.054 ± 0.011 h^−1^, and the average specific cell growth rate during the bioprocess was 0.030 ± 0.01, 0.030 ± 0.005 and 0.024 ± 0.007 h^−1^ respectively (Fig. 4B, Table 3). Likewise, the optimal cell yield on glucose (*Y*_X/S_) and the maximum cell productivity (*P*_X_), that is, which were 4.03 ± 1.25 g g^−1^ and 0.357 ± 0.005 g L^−1^ h^−1^ at pH 7.0, were higher than that at other pH conditions (Table 3). The optimum original pH for maximal cell proliferation of Δ*cpxR* was found to be pH 7.0.

**Table 3:** Parameters of the batch fermentation of Δ*cpxR* at different pH in the 5‐l bioreactor

Parameters	Δ*cpxR*		
	pH 5.5	pH 7.0	pH 8.5
*X*_max_ (g L^−1^)	13.99 ± 1.38 c	20.44 ± 2.65 a	16.12 ± 0.57 b
*P*_max_ (U ml^−1^)	335.71 ± 25.73 b	470.71 ± 15.67 a	340.48 ± 20.36 b
*P*_*X*_ (g L^−1^ h^−1^)	0.231 ± 0.03 b	0.357 ± 0.005 a	0.166 ± 0.02 b
*P*_*P*_ (U ml^−1^ h^−1^)	4.66 ± 1.13 b	6.54 ± 0.58 a	5.67 ± 1.67 ab
*μ* (h^−1^)	0.065 ± 0.013 a	0.065 ± 0.009 a	0.054 ± 0.011 ab
$\bar{\mu }$ (h^−1^)	0.030 ± 0.01 a	0.030 ± 0.005 a	0.024 ± 0.007 b
*q*_*p*_ (U mg^−1^ h^−1^)	18.495 ± 2.56 c	20.540 ± 1.68 b	48.569 ± 4.331 a
$\bar{q_{p}}$ (U mg^−1^ h^−1^)	3.098 ± 0.556 b	3.192 ± 0.796 b	8.392 ± 1.875 a
*Y*_*X/S*_ (g g^−1^)	3.26 ± 0.34 b	4.03 ± 1.25 a	3.74 ± 1.13 ab
*Y*_*P/S*_ (U g^−1^)	78.28 ± 5.30 b	92.79 ± 2.58 a	76.18 ± 6.13 b
*Y*_*P/X*_ (U g^−1^)	24.00 ± 1.09 a	23.03 ± 3.24 a	22.95 ± 0.67 a
*q*_s_(h^−1)^	0.241 ± 0.052 b	0.247 ± 0.007 b	0.314 ± 0.021 a
$\bar{q_{s}}$ (h^−1^)	0.042 ± 0.006 a	0.036 ± 0.013 a	0.057 ± 0.015 a

Data are presented as a mean ± standard deviation from three replicated experiments. Different lowercase letters followed by the data in each row indicate significant differences at *P *=* *0.05.

The influence of different pH on the antibiotic production potential of Δ*cpxR* is portrayed in Fig. 4C. A higher antimicrobial activity was observed at pH 8.5 than that at pH 5.5 and 7.0 during the first 0–18 h. However, after 24 h of bioprocess, the antibiotic activity was significantly increased at neutral pH (pH 7.0) in contrast with pH 5.5 and 8.5. The maximum antibiotic activity (470.71 ± 15.67 U ml^−1^), which was 28.68% and 27.67% higher compared with pH 5.5 and 8.5, was obtained at pH 7.0, where the maximum DCW was obtained. Profiles of the specific antibiotic production rate (*q*_p_) exhibited similar trends at varying pH, and *q*_p_ reached the maximum value at 6 h of the bioprocesses. The highest *q*_*p*_ was achieved at pH 8.5 and reduced by 61.97% and 57.71% at pH 5.5 and 7.0 respectively (Fig. 4D, Table 3). Consistent with the *q*_*p*_ of Δ*cpxR* at different pH, during the bioprocess, the $\bar{q_{p}}$ ofΔ*cpxR* at pH 8.5 was also significantly higher than that at pH 5.5 and 8.5 (Table 3). Similarly, the highest antibiotic yield on glucose (*Y*_P/S_) and the antibiotic productivity (*P*_P_) achieved at pH 7.0 were 92.79 ± 2.58 U g^−1^ and 6.54 ± 0.58 U ml^−1^ h^−1^ respectively. Nevertheless, the maximum antibiotic yield on the cell (*Y*_P/X_) was 24.00 ± 1.09 U g^−1^ at pH 5.5 (Table 3). The findings inferred that among the different pH investigated, pH 7.0 was found to be promising for antibiotic biosynthesis due to the elevated cell growth at this pH.

The influence of varying pH on the biosynthesis of nematophin and Xcns was also determined. A noticeable effect (*p *<* *0.05) of different pH was observed in Xcns biosynthesis. At pH 7.0, Xcn1 production was 5.45‐ and 3.87‐fold higher than that at pH 5.5 and 8.5 respectively (Table 4, Figs S3 and S4). In addition, pH also had a significant effect on nematophin production (Fig. S5). In comparison with pH 5.5 and 7.0, the nematophin concentration was recorded to be significantly higher at pH 8.5. At 72 h, the concentration of nematophin under pH 8.5 was 156.25 ± 3.67 μg ml^−1^, 1.59‐ and 3.69‐fold greater than that at pH 7.0 and 5.5. Likewise, the optimal *Y*_P/S_, *P*_P_. *Y*_P/X_ and *q*_p_ of nematophin at pH 8.5 were 86.71, 58.39, 101.04 and 101.49% higher compared with that at pH 7.0 (Fig. 3B, Table 2).

**Table 4:** The relative amount of xenocoumacins (Xcn1 and Xcn2) in Δ*cpxR* of *Xenorhabdus neamatophila* YL001 at different pH

pH	Relative amount of Xcns^a^	
	Xcn1	Xcn2
5.5	1 c	1 b
7.0	5.45 a	1.06 b
8.5	1.41 b	2.4 a

a The peak area of extracted ion chromatogram (EIC) of Xcns at different pH/the peak area of extracted ion chromatogram (EIC) of Xcns at pH 5.5, the peak area was calibrated by its OD_600_ value, and the relative amount of Xcns at pH 5.5 was referred to 1. Different lowercase letters followed by the data of Xcn1 and Xcn2 at different pH indicate significant differences at *P *=* *0.05.

The rapid growth and rise in biomass at an early phase of fermentation at pH 7.0 resulted in the rapid decrease of DO concentration compared with that at pH 5.5 and 8.5 (Fig. 5). Consistently, glucose uptake at pH 7.0 was faster than that at pH 5.5 and 8.5 (Fig. 4E). Similarly, at the late stage of fermentation, a higher biomass at pH 7.0 resulted in the increased uptake of glucose. During 0–42 h of the fermentation, the total glucose consumption at pH 5.5, 7.0 and 8.5 was 76.0, 87.67 and 69.67% respectively. At the end of the fermentation, the glucose concentration was 1.71, 0.93 and 1.69 g L^−1^ at pH 5.5, 7.0 and 8.5 respectively. However, the highest *q*_*s*_ (0.314 ± 0.021 h^−1^) was obtained at pH 8.5 and decreased by 30.29% and 27.13% at pH 5.5 and 7.0 respectively (Fig. 4D). The average specific glucose consumption rate during the bioprocess at pH 5.5, 7.0 and 8.5 was 0.042 ± 0.006, 0.036 ± 0.013 and 0.057 ± 0.015 h^−1^ respectively (Table 3).

### Batch fermentation characteristics of Δ*cpxR* mutation in the 70‐l bioreactor

**Figure 6:**
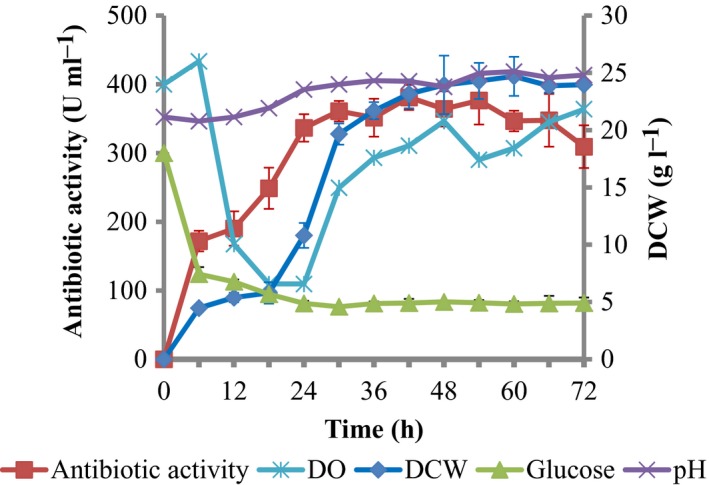
Time‐course data of batch fermentation of Δ*cpxR* mutant of *Xenorhabdus nematophila* YL001 in 70‐l bioreactor with the initial pH of 7.0, the agitation speed of 150 rpm and the aeration rate of 40.0 L min^−1^.

Based on the 5‐l bioreactor‐based fermentation results, the batch fermentation kinetics of Δ*cpxR* mutation was carried out in the 70‐l bioreactor. The growth profile displayed a distinctive exponential and stationary phase, and antibiotic production occurred throughout the bioprocess (Fig. 6). The maximum DCW and antibiotic activity (*P*_max_) in 70‐l bioreactor were 24.71 ± 2.31 g L^−1^ and 381.07 ± 15.74 U ml^−1^, which was increased by 18.97%, and decreased by 27.71% compared to that in 5‐l bioreactor respectively. The concentration of nematophin (72.85 ± 2.58 μg ml^−1^) produced by Δ*cpxR* at 72 h was decreased by 26.06% compared to the 5‐l bioreactor. *Y*_P/S_, *P*_P_. *Y*_P/X_ and *q*_p_ of nematophin in 70‐l bioreactor were decreased by 14.01, 26.28, 36.93 and 37.31% compared with that in the 5‐l bioreactor (Fig. 3C, Table 2). Xcn1 of Δ*cpxR* was decreased by 11.0% relative to that in the 5‐l bioreactor (data not shown).

## Discussion

In contrast to the wildtype strain, Δ*cpxR* mutant exhibited a prolonged lag phase, and lower DCW at early‐ and mid‐stage. These findings were consistent with the previous report, in which *cpxR* inactivation results in a prolonged lag phase when cultivated in either LB culture or haemolymph (Herbert *et al*., 2007). At the late stage of fermentation, the DCW of Δ*cpxR* was enhanced relative to the wildtype, whereas, in the previous study, the cell biomass between a *cpxR* deletion mutant and wildtype of *X. nematophila* had no significant difference (Herbert *et al*., 2007). The difference in growth profiles between the parent strain and Δ*cpxR* derivative may be associated with the rapid adaptability to fluctuating environmental perturbations. CpxRA could sense the changing environment and regulate the growth and metabolisms to adapt the environment of insect haemolymph (Herbert *et al*., 2007; Tran and Goodrich‐Blair, 2009; Herbert *et al*., 2009). However, Δ*cpxR* mutant was defective in adapting to this nutrient source and displayed a protracted lag phase. This may be due to the lack of metabolism adaptability of Δ*cpxR1* mutant to the nutrients available in haemolymph but is more prone to insect antimicrobial activities (Herbert *et al*., 2007). Previously, it has been found that the Δ*cpxR1* exhibited a prolonged lag phase during *in‐vitro* growth in haemolymph (Herbert *et al*., 2007), a finding consistent with the idea that it is defective in acclimatizing to this nutrient source.

Generally, antibiotic biosynthesis is induced at high cell density and is regulated, in most of the cases by quorum sensing (Romero *et al*., 2011). In this study, in all cases, the nematophin biosynthesis could be improved by raising the concentration of antibiotic‐producing cells (Fig. 3). Li *et al*. (1997) and Park *et al*. (2009) reported comparable outcomes that the concentration of nematophin and Xcns increased by increasing the bacterial cell density and attained the maximal level at the stationary phase. However, in Δ*cpxR* mutant, the level of Xcn1 was increased significantly while nematophin was decreased during the stationary phase, where the DCW of Δ*cpxR* was higher. Our findings confirmed that quorum sensing is not an essential process to regulate the antimicrobial biosynthesis (Singh and Forst, 2016), and global regulators particularly govern the biosynthesis of small molecules in *X. nematophila* (Park *et al*., 2009; Engel *et al*., 2017). Therefore, identifying and engineering of these regulators can provide a noteworthy approach for the discovery of new secondary metabolites and increased production of useful molecules.

In this study, the Δ*cpxR* strain exhibited an increase in antibiotic activity. This suggested that the biosynthetic pathway of secondary metabolites was induced in Δ*cpxR*. However, although the production of Xcn1 in Δ*cpxR* was 3.07‐fold higher, nematophin production was significantly decreased. Meanwhile, we did not rule out the possibilities that the *cpxR* gene disruption could induce the production of previously cryptic metabolites, and the biological activities of these induced cryptic metabolites need to be further validated by purification and characterization. Taken together, the improved antibiotic activity of Δ*cpxR* might be attributed to the mutual effect of higher Xcn1 or the cryptic antibiotics produced in Δ*cpxR* strain.

In CpxRA signal transduction system, CpxA senses a variety of stresses and changes in external conditions, including pH, phosphorylates CpxR, and activates this response regulator to regulate the expression of its regulon (Herbert *et al*., 2007). Previously, we have demonstrated the significant influence of different pH on the growth profile and antibiotic biosynthesis titre of *X. nematophila* YL001 (Wang *et al*., 2011). However, it is uncertain whether CpxA can sense the pH changing and affect the growth and antibiotic production of *cpxR*‐inactivated mutant. Our study indicated that pH had a marked consequence of the growth and production of antibiotic by Δ*cpxR* strain. The cell biomass, the maximum DCW and antibiotic production of Δ*cpxR* strain all were varied at different pH conditions. However, there was no significant difference in cell biomass of the wildtype at different pH conditions (Wang *et al*., 2011). The growth difference between the wildtype and Δ*cpxR* strain might be related to the hypersensitivity of Δ*cpxR* strain to alkaline conditions. Danese and Silhavy (1998) found that the *cpxR* null mutant of *E. coli* was the most hypersensitive to alkaline condition, and showed marked growth defects at an extreme alkaline pH. Also, the antibiotic production of Δ*cpxR* strain had a significant difference between pH 5.5, 7.0 and 8.5 (Table 1, Fig. S3). Our previous study revealed that antibiotic activity and the relative amount of Xcn1 of wildtype at pH 8.5 were higher relative to that at pH 5.5 and pH 7.0 (Guo, 2015). Moreover, in *X. nematophila*, CpxR and OmpR negatively regulate the production of Xcn1 (Park *et al*., 2009; Tang, 2015). Although CpxR can be activated by alkaline pH, it works as a CpxA‐dependent response (Siryaporn and Goulian, 2008; Wolfe *et al*., 2008). At pH 8.5, the expression level of *cpxR* and *ompR* of wildtype of *X. nematophila* YL001 was significantly lower than that at pH 5.5 and 7.0 (Guo, 2015). Therefore, the Xcn1 levels of the wildtype were increased at pH 8.5. However, the *ompR* expression level of Δ*cpxR* strain at pH 8.5 was significantly greater than that at pH 5.5 and 7.0 (Tang, 2015). Thus, at pH 7.0 the Δ*cpxR* strain exhibited the higher levels of Xcn1 and antibiotic activity. The ability of Δ*cpxR* strain to sense the changes in pH, and regulating the growth and antibiotic production might be related to the cross‐talk between a histidine kinase and a non‐cognate response regulator of different two‐component signalling systems. Siryaporn and Goulian (2008) detected the cross‐talk between the histidine kinase CpxA and non‐cognate response regulator OmpR in *E. coli*. Hence, we speculated that OmpR of Δ*cpxR* strain could be triggered by alkaline pH conditions.

Based on the scale‐up method of a constant mass transfer coefficient, *X. nematophila* YL001 was successfully scaled‐up from shaking flask to a 5‐l bioreactor, and further to a 70‐l bioreactor (Wang, 2004). In this study, according to the fermentation condition of *X. nematophila* YL001 in a 70‐l bioreactor, Δ*cpxR* strain was cultured in a 70‐l bioreactor. Δ*cpxR* strain exhibited the similar kinetics profiles, compared with fermentation in the 5‐l bioreactor. However, the profiles of DO concentration of Δ*cpxR* strain in 5‐ and 70‐l bioreactor had a significant difference (*p *<* *0.05, *t *=* *5.491, *d*_f_ = 16). In the 70‐l bioreactor, the DO concentration was above 20% during the whole bioprocess but was maintained to around zero level in a growth phase in the 5‐l bioreactor. The fermentation condition in 70‐l bioreactor could ensure the sufficient supply of oxygen in the fermentation process that is favourable to both the cell growth and metabolic activity. So, the maximum DCW of Δ*cpxR* strain in the 70‐l bioreactor was increased by 18.97%, but the maximum antibiotic activity was decreased by 27.71%, compared to that in the 5‐l bioreactor. These findings were consistent with the results of scale‐up of *X. nematophila* YL001 from a 5‐l to the 70‐l bioreactor. These showed that the fermentation conditions used in the 70‐l bioreactor were beneficial for cell growth, but not favourable to the biosynthesis of antibiotics. Thus, the optimum fermentation condition for antibiotic production by Δ*cpxR* strain will be further investigated.

These findings confirmed that Δ*cpxR* mutation significantly increased the biomass of *X. nematophila* at a late stage of fermentation, and promoted the production of Xcns, but decreased the production of nematophin. In addition, pH had remarkable effects on the bacterial cell growth and antimicrobial activity of Δ*cpxR* mutant. Thus, this work provides an efficient way to obtain massive antimicrobial production of Xcns or nematophin in *X. nematophila*.

## Experimental procedures

### Microorganism

The *X. nematophila* wildtype strain was isolated from the soil in Yangling, China. The *cpxR*‐null mutant strain (Δ*cpxR*) derived from YL001 by the allelic exchange that contains a kanamycin (Km) cassette instead of the *cpxR* gene was used (Tang, 2015). Phase I variant of *X. nematophila* preserved in glycerol at −70°C was used as seed inoculum for cultures. NBTA NA medium supplemented with triphenyl tetrazolium chloride (0.040 g L^−1^) and bromothymol blue (0.025 g L^−1^) was employed to test the phase variant of *X. nematophila*.

### Preparation of inoculum

A loopful of phase I of *X. nematophila* YL001 wildtype and Δ*cpxR* was inoculated into 250‐ml Erlenmeyer flasks comprising 100 ml sterilized NB (NA without agar) medium with the final pH of 7.2. The flasks were then incubated in dark at 28°C in a temperature‐controlled rotary shaker at an agitation speed of 150 rpm for 16 to 24 h.

### Batch fermentation characteristics in the laboratory‐scale bioreactor

Fermentation of Δ*cpxR* of *X. nematophila* YL001 was investigated in batch fermentation using a 5‐l bioreactor (Eastbio, China). The pH, temperature, DO and agitation speed were monitored online by a computer‐coupled system. The medium containing (g L^−1^) glucose 6.13, peptone 21.29, KH_2_PO_4_ 0.86, MgSO_4_∙7H_2_O 1.50, Na_2_SO_4_ 1.72, K_2_HPO_4_ 1.11 and (NH_4_)_2_SO_4_ 2.46 was adjusted to pH 7.2 by the addition 1 M HCl or 1 M NaOH. The influence of varying pH on the growth pattern and antibiotic production of Δ*cpxR* was also evaluated in batch fermentation using the same bioreactor. Details have been shown in the Supporting Information.

### Batch fermentation process in 70‐l bioreactor

Fermentation of Δ*cpxR* was also carried out in a 70‐l bioreactor (Eastbio, China) with a working volume of 50 L. The bioreactor was equipped with two six‐blade disc turbine impeller. The pH, temperature, DO and agitation speed were monitored online. The fermentation medium, pH, temperature and inoculum level used were identical with the 5‐l bioreactor. Throughout the fermentation bioprocessing, the temperature levels were regulated automatically. The bioreactors were operated under well‐controlled conditions as mentioned above for 5‐l bioreactor for 72 h.

### Analytical methods

The method for the determination of DCW (g L^−1^), the specific cell growth rate (*μ*), the glucose concentration and the antibiotic activity assay was shown in Supporting Information. All of the results were averaged and presented as the mean ± standard deviation from triplicate experiments. Student's t‐test using SPSS 18.0 (SPSS, Chicago, IL, USA) statistically analysed the data.

## Conflict of interest

The authors have no conflict of interest.

## Author Contributions

Y.H.W. proposed and supervised the project. X.Z. designed the experiments. S.Q.G., Z.Y.W., B.L.L., J.T.G., X.L.F. and Q.T. conducted the experiments. S.Q.G., M.B., X.L.F. and Y.H.W. analysed the data and drafted the manuscript. All authors read and approved the final manuscript.

## References

[mbt213362-bib-0001] Akhurst, R.J. (1982) Antibiotic activity of *Xenorhabdus* spp., bacteria symbiotically associated with insect pathogenic nematodes of the families *Heterorhabditidae* and *Steinernematidae* . J Gen Microbiol 128: 3061–3065. 7183749 10.1099/00221287-128-12-3061

[mbt213362-bib-0002] Bozhüyük, K. A. J. , Zhou, Q. , Engel, Y. , Heinrich, A. , Pérez, A. , and Bode, H. B. (2016) Biosynthesis of the antibiotic nematophin and its elongated derivatives in entomopathogenic bacteria In The Molecular Biology of Photorhabdus Bacteria. Cham: Springer, pp. 59–79.

[mbt213362-bib-0003] Cai, X. , Challinor, V.L. , Zhao, L. , Reimer, D. , Adihou, H. , Grün, P. , *et al* (2017) Biosynthesis of the antibiotic nematophin and its elongated derivatives in entomopathogenic bacteria. Org Lett 19: 806–809. 28134534 10.1021/acs.orglett.6b03796

[mbt213362-bib-0004] Challinor, V.L. , and Bode, H.B. (2015) Bioactive natural products from novel microbial sources. Ann N Y Acad Sci 1354: 82–97. 26509922 10.1111/nyas.12954

[mbt213362-bib-0005] Chaston, J.M. , Suen, G. , Tucker, S.L. , Andersen, A.W. , Bhasin, A. , Bode, E. , *et al* (2011) The entomopathogenic bacterial endosymbionts *Xenorhabdus* and *Photorhabdus*: Convergent Lifestyles from Divergent Genomes. PLoS ONE 6: e27909. 22125637 10.1371/journal.pone.0027909 PMC3220699

[mbt213362-bib-0006] Chen, G. , Dunphy, G.B. , and Webster, J.M. (1994) Antifungal activity of two *Xenorhabdus* species and *Photorhabdus luminescens*, bacteria associated with the nematodes *Steinernema* species and *Heterorhabditis megidis* . Biol Control 4: 157–162.

[mbt213362-bib-0007] Chen, G. , Maxwell, P. , Dunyhy, G.B. , and Webster, J.M. (1996) Culture conditions for *Xenorhabdus* and *Photorhabdus* symbionts of entomopathogenic nematodes. Nematologica 42: 124–130.

[mbt213362-bib-0009] Crawford, J.M. , Portmann, C. , Kontnik, R. , Walsh, C.T. , and Clardy, J. (2011) NRPS substrate promiscuity diversifies the Xenematides. Org Lett 13: 5144–5147. 21888371 10.1021/ol2020237 PMC3184645

[mbt213362-bib-0010] Danese, P.N. , and Silhavy, T.J. (1998) CpxP, a stress‐combative member of the Cpx regulon. J Bacteriol 180: 831–839. 9473036 10.1128/jb.180.4.831-839.1998 PMC106961

[mbt213362-bib-0011] Engel, Y. , Windhorst, C. , Lu, X. , Goodrich‐Blair, H. and Bode, H.B. . (2017) The global regulators Lrp, LeuO, and HexA control secondary metabolism in entomopathogenic bacteria. Front Microbiol 8: 209 10.3389/fmicb.2017.00209. 28261170 PMC5313471

[mbt213362-bib-0012] Forst, S. and Nealson, K. . (1996) Molecular biology of the symbiotic‐pathogenic bacteria *Xenorhabdus* spp. and *Photorhabdus* spp. Microbiol Rev 60: 21. 8852894 10.1128/mr.60.1.21-43.1996 PMC239416

[mbt213362-bib-0013] Goodrich‐Blair, H. , and Clarke, D.J. (2007) Mutualism and pathogenesis in *Xenorhabdus* and *Photorhabdus*: two roads to the same destination. Mol Microbiol 64: 260–268. 17493120 10.1111/j.1365-2958.2007.05671.x

[mbt213362-bib-0014] Guo, S.Q. (2015) Study on Antibacterial Mechanism of Xenorhabdus Nematophila Regulated by pH. China: Northwest A&F University.

[mbt213362-bib-0015] Herbert, E.E. , and Goodrich‐Blair, H. (2007) Friend and foe: the two faces of *Xenorhabdus nematophila* . Nat Rev Microbiol 5: 634–646. 17618298 10.1038/nrmicro1706

[mbt213362-bib-0016] Herbert, E.E. , Cowles, K.N. , and Goodrich‐Blair, H. (2007) CpxRA regulates mutualism and pathogenesis in *Xenorhabdus nematophila* . Appl Environ Microbiol 73: 7826–7836. 17951441 10.1128/AEM.01586-07 PMC2168154

[mbt213362-bib-0017] Herbert, E.E. , Andersen, A.W. , and Goodrich‐Blair, H. (2009) CpxRA influences *Xenorhabdus nematophila* colonization initiation and outgrowth in *Steinernema carpocapsae* nematodes through regulation of the *nil* locus. Appl Environ Microbiol 75: 4007–4014. 19376901 10.1128/AEM.02658-08 PMC2698367

[mbt213362-bib-0018] Hinchliffe, S.J. (2010) Insecticidal toxins from the *Photorhabdus* and *Xenorhabdus* bacteria. Open Toxinol J 3: 101–118.

[mbt213362-bib-0019] Huang, W.R. , Zhu, C.X. , Yang, X.F. , Yang, H.W. , Xu, H.Z. , Xie, Y.Y. , and Jian, H. (2005) Isolation and structural identification of main component CB6‐1 produced by *Xenorhabdus nematophilus* var.pekingensis. Chin J Antibiot 30: 513–515.

[mbt213362-bib-0020] Huang, W.R. , Yang, X.F. , Yang, H.W. , Liu, Z. , and Yuan, J.J. (2006) Identification and activity of a antibacterial substance from *Xenorhabdus nematophila* var. Pekingense. Nat Prod Res Dev 18: 25–28.

[mbt213362-bib-0021] Ji, D. , Yi, Y. , Kang, G.H. , Choi, Y.H. , Kim, P. , Baek, N.I. , and Kim, Y. (2004) Identification of an antibacterial compound, benzylideneacetone, from *Xenorhabdus nematophila* against major plant‐pathogenic bacteria. FEMS Microbiol Lett 239: 241–248. 15476972 10.1016/j.femsle.2004.08.041

[mbt213362-bib-0022] Kennedy, G. , Viziano, M. , Winders, J.A. , Cavallini, P. , Gevi, M. , Micheli, F. , *et al* (2000) Studies on the novel anti‐staphyloccal compound nematophin. Bioorg Med Chem Lett 10: 1751–1754. 10937740 10.1016/s0960-894x(00)00331-0

[mbt213362-bib-0023] Li, J. , Chen, G. , Webster, J.M. , and Czyzewska, E. (1995) Antimicrobial metabolites from a bacterial symbiont. J Nat Prod 58: 1081–1086. 7561900 10.1021/np50121a016

[mbt213362-bib-0024] Li, J. , Chen, G. , and Webster, J.M. (1997) Nematophin, a novel antimicrobial substance produced by *Xenorhabdus nematophilus* (Enterobactereaceae). Can J Microbiol 43: 770–773. 9304787 10.1139/m97-110

[mbt213362-bib-0025] McInerney, B.V. , Taylor, W.C. , Lacey, M.J. , Akhurst, R.J. , and Gregson, R.P. (1991) Biologically active metabolites from *Xenorhabdus* Spp., Part 2. Benzopyran‐1‐one derivatives with gastroprotective activity. J Nat Prod 54: 785–795. 1955881 10.1021/np50075a006

[mbt213362-bib-0027] Morgan, J.A. , Sergeant, M. , Ellis, D. , Ousley, M. , and Jarrett, P. (2001) Sequence analysis of insecticidal genes from *Xenorhabdus nematophilus* PMFI296. Appl Environ Microbiol 67: 2062–2069. 11319082 10.1128/AEM.67.5.2062-2069.2001 PMC92837

[mbt213362-bib-0028] Park, D. , and Forst, S. (2006) Co‐regulation of motility, exoenzyme and antibiotic production by the EnvZ‐OmpR‐FlhDC‐FliA pathway in *Xenorhabdus nematophila* . Mol Microbiol 61: 1397–1412. 16889644 10.1111/j.1365-2958.2006.05320.x

[mbt213362-bib-0029] Park, D. , Ciezki, K. , van der Hoeven, R. , Singh, S. , Reimer, D. , Bode, H.B. , and Forst, S. (2009) Genetic analysis of xenocoumacin antibiotic production in the mutualistic bacterium *Xenorhabdus nematophila* . Mol Microbiol 73: 938–949. 19682255 10.1111/j.1365-2958.2009.06817.x

[mbt213362-bib-0030] Paul, V.J. , Frautschy, S. , Fenical, W. , and Nealson, K.H. (1981) Antibiotics in microbial ecology. J Chem Ecol 7: 589–597. 24420598 10.1007/BF00987707

[mbt213362-bib-0031] Romero, D. , Traxler, M.F. , López, D. , and Kolter, R. (2011) Antibiotics as signal molecules. Chem Rev 111: 5492–5505. 21786783 10.1021/cr2000509 PMC3173521

[mbt213362-bib-0032] Sergeant, M. , Jarrett, P. , Ousley, M. , and Morgan, J.A. (2003) Interactions of insecticidal toxin gene products from *Xenorhabdus nematophilus* PMFI296. Appl Environ Microbiol 69: 3344–3349. 12788735 10.1128/AEM.69.6.3344-3349.2003 PMC161543

[mbt213362-bib-0033] Sheets, J.J. , Hey, T.D. , Fencil, K.J. , Burton, S.L. , Ni, W. , Lang, A.E. , *et al* (2011) Insecticidal toxin complex proteins from *Xenorhabdus nematophilus*: structure and pore formation. J Biol Chem 286: 22742–22749. 21527640 10.1074/jbc.M111.227009 PMC3123041

[mbt213362-bib-0034] Singh, S. and Forst, S. . (2016) Antimicrobials and the natural biology of a bacterial‐nematode symbiosis In The Mechanistic Benefits of Microbial Symbionts. Cham: Springer, pp. 101–119.

[mbt213362-bib-0035] Siryaporn, A. , and Goulian, M. (2008) Cross‐talk suppression between the CpxA‐CpxR and EnvZ‐OmpR two‐component systems in *E.coli* . Mol Microbiol 70: 494–506. 18761686 10.1111/j.1365-2958.2008.06426.x PMC2761842

[mbt213362-bib-0036] Sundar, L. , and Chang, F.N. (1993) Antimicrobial activity and biosynthesis of indole antibiotics produced by *Xenorhabdus nematophilus* . J Gen Microbiol 139: 3139–3148. 7510325 10.1099/00221287-139-12-3139

[mbt213362-bib-0037] Tang, Q. (2015) Effect of CpxR Gene Knockout on the Antibacterial Activity and Metabolism of Xenorhabdus Nematophila. China: Northwest A&F University.

[mbt213362-bib-0038] Thomas, G.M. , and Poinar, G.O. (1979) *Xenorhabdus* gen. nov., a genus of entomopathogenic, nematophilic bacteria of the family enterobacteriaceae. Int J Syst Bacteriol 29: 352–360.

[mbt213362-bib-0039] Tobias, N.J. , Wolff, H. , Djahanschiri, B. , Grundmann, F. , Kronenwerth, M. , Shi, Y.M. , *et al* (2017) Natural product diversity associated with the nematode symbionts *Photorhabdus* and *Xenorhabdus* . Nat Microbiol 2: 1676–1685. 28993611 10.1038/s41564-017-0039-9

[mbt213362-bib-0040] Tran, E.E.H. , and Goodrich‐Blair, H. (2009) CpxRA contributes to *Xenorhabdus nematophila* virulence through regulation of *lrhA* and modulation of insect immunity. Appl Environ Microbiol 75: 3998–4006. 19376911 10.1128/AEM.02657-08 PMC2698343

[mbt213362-bib-0041] Wang, Y.H. (2004) Studies on the Bioactivity and Culture of Bacterial Symbiont of Entomopathogenic Nematodes. China: Northwest A&F University.

[mbt213362-bib-0042] Wang, Y.H. , Feng, J.T. , Zhang, Q. , and Zhang, X. (2008a) Optimization of fermentation condition for antibiotic production by *Xenorhabdus nematophila* with response surface methodology. J Appl Microbiol 104: 735–744. 17953686 10.1111/j.1365-2672.2007.03599.x

[mbt213362-bib-0043] Wang, Y.H. , Li, Y.P. , Zhang, Q. , and Zhang, X. (2008b) Enhanced antibiotic activity of *Xenorhabdus nematophila* by medium optimization. Bioresource TechnoL 99: 1708. 10.1016/j.biortech.2007.03.053 17531470

[mbt213362-bib-0044] Wang, Y.H. , Fang, X.L. , Li, Y.P. , and Zhang, X. (2010) Effects of constant and shifting dissolved oxygen concentration on the growth and antibiotic activity of *Xenorhabdus nematophila* . Bioresource Technol 101: 7529–7536. 10.1016/j.biortech.2010.04.070 20488698

[mbt213362-bib-0045] Wang, Y. , Fang, X. , Cheng, Y. , and Xing, Z. (2011) Manipulation of pH shift to enhance the growth and antibiotic activity of *Xenorhabdus nematophila* . J Biomed Biotechnol 2011: 672369. 21660139 10.1155/2011/672369 PMC3110314

[mbt213362-bib-0046] Wolfe, A.J. , Bruno, N.P. , Lima, P. , and Zemaitaitis, B. (2008) Signal Integration by the two‐component signal transduction response regulator CpxR. J Becteriol 190: 2314–2322. 10.1128/JB.01906-07 PMC2293188 18223085

[mbt213362-bib-0047] Yang, X.F. , Qiu, D.W. , Zhang, Y.L. , Zeng, H.M. , Liu, Z. , Yang, H.W. , *et al* (2009) A toxin protein from *Xenorhabdus nematophila* var. pekingensis and insecticidal activity against larvae of *Helicoverpa armigera* . Biocontrol Sci Techn 19: 943–955.

[mbt213362-bib-0048] Yang, X. , Qiu, D. , Yang, H. , Liu, Z. , Zeng, H. , and Yuan, J. (2011) Antifungal activity of xenocoumacin 1 from *Xenorhabdus nematophilus* var. pekingensis against *Phytophthora infestans* . World J Microb Biot 27: 523–528.

[mbt213362-bib-0049] Zhou, T. , Yang, X. , Qiu, D. , and Zeng, H. (2017) Inhibitory effects of xenocoumacin 1 on the different stages of *Phytophthora capsici* and its control effect on *Phytophthora blight* of pepper. Biocontrol 62: 1–10.

